# Cemented versus uncemented total hip replacement for femoral neck fractures in elderly patients: a retrospective, multicentre study with a mean 5-year follow-up

**DOI:** 10.1186/s13018-020-01980-4

**Published:** 2020-09-30

**Authors:** Shuai Mao, Baomin Chen, Ying Zhu, Liang Qian, Jinluan Lin, Xinchao Zhang, Weiguang Yu, Guowei Han

**Affiliations:** 1 Department of Hepatobiliary Surgery, The First Affiliated Hospital, Sun Yat-sen University, No. 58, Zhongshan 2nd Road, Yuexiu District, Guangzhou, 510080 China; 2 Department of Radiology, The First Affiliated Hospital, Sun Yat-sen University, No. 58, Zhongshan 2nd Road, Yuexiu District, Guangzhou, 510080 China; 3 Department of Anesthesiology, The Seventh Affiliated Hospital, Sun Yat-sen University, No. 628, Zhenyuan Road, Guangming New District, Shenzhen, 518107 China; 4 Department of Orthopaedics, The Affiliated Hospital of Fujian Medical University, Chazhong Road No. 20, Taijiang District, Fuzhou, 350005 Fujian China; 5 Department of Orthopedics, Jinshan Hospital, Fudan University, Longhang Road No. 1508, Jinshan District, Shanghai, 201508 China; 6 Department of Orthopedics, The First Affiliated Hospital, Sun Yat-sen University, No. 58, Zhongshan 2nd Road, Yuexiu District, Guangzhou, 510080 China

**Keywords:** Cemented, Uncemented, Total hip replacement, Femoral neck fracture, Prosthesis revision

## Abstract

**Background:**

Cemented or uncemented total hip replacement (CTR or UTR) for femoral neck fractures (AO/OTA type 31B/C) is a relatively common procedure in elderly individuals. The recent literature is limited regarding long-term outcomes following CTR versus UTR in the Asian population.

**Methods:**

Using our institutional database, we performed long-term outcome analysis on 268 patients with femoral neck fractures (AO/OTA type 31B/C) who had undergone a primary UTR or CTR (CTR: *n* = 132, mean age, 67.43 ± 6.51 years; UTR: *n* = 136, mean age, 67.65 ± 6.13 years) during 2007–2014, and these patients were followed until 2019. Follow-up occurred 1, 3, 6, and 12 months postoperatively and yearly thereafter. The primary endpoint was the Harris hip score (HHS); the secondary endpoint was the incidence of orthopaedic complications.

**Results:**

The mean follow-up time was 62.5 months (range, 50.1–76.1 months). At the final follow-up, the HHS was 79.39 ± 16.92 vs 74.18 ± 17.55 (CTR vs UTR, respectively, *p* = 0.011). Between-group significant differences were observed regarding the incidence of prosthesis revision, prosthesis loosening, and periprosthetic fracture (7.6% [95% CI, 6.4–8.2] for CTR vs 16.9% [95% CI, 14.7–17.3] for UTR, *p* = 0.020; 9.8% [95% CI, 8.3–10.7] for CTR vs 19.9% [95% CI, 18.2–20.9] for UTR, *p* = 0.022; 5.3% [95% CI, 4.4–6.7] for CTR vs 13.2% [95% CI, 12.1–13.8] for UTR, *p* = 0.026, respectively).

**Conclusion:**

CTR showed superiority to UTR by improving the HHS and decreasing the incidence of orthopaedic complications. Our findings need to be confirmed in a prospective, randomized controlled study to verify whether they can be applicable to a broader population.

## Background

Femoral neck fractures (FNFs) in elderly individuals are associated with impaired mobility, loss of independence, and an overabundance of morbidity and mortality [1–3]. With the reversal of the ageing pyramid and the increasing prevalence of osteoporosis, FNFs remain a public health concern [3–5]. Treatment for FNFs remains challenging and controversial [3]. Cemented or uncemented total hip replacement (CTR or UTR) remains a widely accepted method for hip replacement after fracture [6]. Promising results have been described for patients with FNFs treated with a CTR or UTR [7–9]. Most of these studies, however, have been from a highly specialized medical institution and have introduced only one specific brand of instrument [7]. Additionally, few register-based studies have described long-term outcomes following CTR or UTR in individuals at a population-based level [3, 7]. In addition, the features of high selectivity and a short length of follow-up are highly common in previous retrospective studies [5, 10]. Consequently, the findings in these previous reports do not seem to reflect the actual situation. Indecision as to which type of endoprosthesis is optimal (CTR or UTR) for the treatment of FNFs in elderly patients results in noteworthy variation in the use of each intervention internationally. Several studies have indicated the benefits of UTR over CTR with respect to complication rates and operation times [11–13]. The remaining concerns are that the long-term outcomes of UTR are not as robust as those of CTR, that UTR does not decrease the need for early revision (less than 5 years), and that UTR requires excessive intervention to uphold conventional hip function [6, 14]. For UTR, the risks of early prosthesis revision or excessive interventions, as well as the disproportionate expense associated with these procedures, may offset some of its initial benefits [13, 15].

In our study, we used the data from the First Affiliated Hospital, Sun Yat-sen University; the Seventh Affiliated Hospital, Sun Yat-sen University; the Affiliated Hospital of Fujian Medical University; and Jinshan Hospital, Fudan University, to compare long-term outcomes for Asian patients with FNFs (AO/OTA type 31B/C) who had undergone a primary CTR or UTR.

## Materials and methods

### Study population

The data collected contains information on FNF characteristics, approaches, implant type, and follow-up results and data, with scheduled visits at 1, 3, 6, and 12 months postoperatively and yearly thereafter. Data covering the period of November 1, 2007, to August 31, 2014, with follow-up to November 2019, were obtained from the 4 tertiary medical institutions. The registry records of all patients who had undergone a primary unilateral CTR or UTR during the study period with a principle diagnosis of FNF (AO/OTA type 31B/C) were retrospectively analysed. Primary FNF (AO/OTA type 31B/C) was considered the indication for these surgeries. The design used for CTR was the Exeter Universal stem combined with the All-poly cup (Stryker, Mahwah, NJ). The design used for UTR was the Taperloc stem (Biomet, Warsaw, IN) combined with a ram-extruded bar stock polyethylene cup (GUR 415; Hoechst Celanese Corp, Houston, TX). The surgical procedure was performed according to the manufacturers’ instructions. A direct anterior approach was used for all patients. The technical details and rehabilitation instructions have been described in our previous report [16]. The patient inclusion criteria were age 60 years or older and FNFs (AO/OTA type 31B/C) confirmed using conventional radiographs or computed tomography (CT). The main exclusion criteria were poor clinical data, multiple fractures, prior hip problems (i.e. osteoarthritis, arthroplasty, neurologic dysfunction, and distinct bone loss), pathological fractures or metastatic diseases, non-dependent living conditions, an injury severity score (ISS) ≥ 10, severe medical diseases (i.e. hypertension with complications, hyperthyroidism, diabetes with a history of frequent hypoglycaemic events, chronic obstructive pulmonary disease [COPD] identified in accordance with the Global Initiative for COPD guidelines and having at least one International Classification of Disease, Tenth Revision [ICD-10, codes J41-J44], and organ failure [heart failure, renal failure, or liver failure]), non-healing wounds, a history of dependence on alcohol or opioids, a body mass index (BMI) > 45 kg/m^2^, hypoalbuminaemia (<25 g/L), inability to consent to the instructions, motor neurone disease, vascular cognitive impairment [17], and an American Society of Anesthesiologists (ASA) score of IV or V. The ICD-10-Chinese Modification codes were used to identify the major disease conditions. Because this study was a retrospective study, the timing of the follow-up assessments was conducted per each institution’s standard of care. The primary endpoint was the Harris hip score (HHS), which was applied to determine the functional level and to evaluate pain; the secondary endpoint was the rate of orthopaedic complications.

Prophylactic antibiotics with cefazolin 2.0 g (China Resources, Shenzhen, China) was given twice a day for 3 days, starting the day prior to surgery. Patients were mobilized with early full weight-bearing ambulation after surgery with the aid of a four-legged armrest as tolerated. After 1–2 months of surgery, full weight-bearing ambulation was encouraged without restriction.

### Method of assessment

All patients were reviewed retrospectively by two independent assessors (SM and BC). Image data were confirmed independently by two of the authors (YZ and LQ). The primary endpoint was the HHS (range, 0–100), with higher scores indicating better function. The secondary endpoint was the incidence of orthopaedic complications, mainly concerning implant-related orthopaedic events (prosthesis revision, loosening, periprosthetic fracture, dislocation, neurologic injury [temporary or permanent], insufferable hip pain, thrombotic events, and heterotopic ossification).

### Statistical analysis

The descriptive statistics of the subjects are reported as the means ± standard deviations (SDs), medians, or interquartile ranges (IQRs) for continuous variables and as frequencies for categorical variables. Follow-up time was defined as the time from the date of primary CTR or UTR to the date of either death from any cause or the final follow-up, whichever occurred first. The Kaplan–Meier method was used to estimate survival, and the log-rank test was used to compare the survival curves. Loosening of the acetabulum component observed via radiography was defined as migration of > 2 mm or a large radiolucent zone around the acetabulum component [18]. Loosening of the stem on radiography was defined as axial subsidence > 2 mm, varus inclination of the stem of > 3, or continuous new radiolucent lines developing around the proximal 2/3 of the implant [9, 19, 20]. For the secondary endpoint, a competing risk approach was conducted to assess the cumulative incidence for each event, which enabled us to discriminate between-group events. Heterotopic ossification was judged using the Brooker classification system. Prosthesis revision was defined as a revision of arthroplasty from any cause [10]. Stress shielding (SS) was determined by the classification of Engh et al. [21]. Differences in continuous variables (i.e. the HHS) between groups were analysed with the independent samples *T* test; differences in categorical data were compared with chi-squared tests. All the statistical tests were two-sided, and the level of significance was set at *p* = 0.05. Data manipulation and analysis were performed with SPSS software version 24 (IBM Corp., Armonk, NY).

## Results

**Fig. 1:**
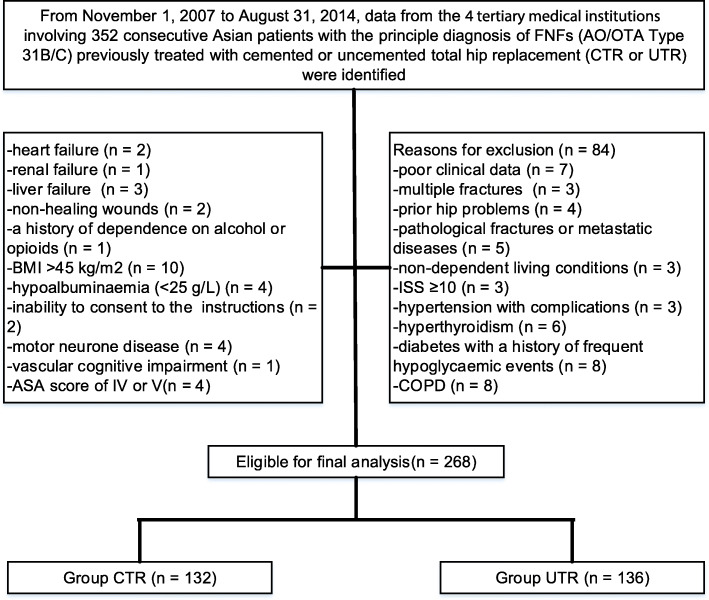
Flow diagram demonstrating methods for identification and exclusion of studies comparing the long-term outcomes of patients with femoral neck fractures (AO/OTA type 31B/C) treated with primary unilateral cemented or uncemented total hip replacement (CTR or UTR, respectively)

In total, 268 consecutive patients (268 hips) were identified from the registry with a mean of 5 years of follow-up (CTR: *n* = 132, mean age, 67.43 ± 6.51 years; UTR: *n* = 136, mean age, 67.65 ± 6.13 years). All CTRs or UTRs were carried out at four different medical institutions by 12 surgeons who all had experience with arthroplasty. At the end of the analysis of follow-up data, the mean follow-up time was 62 months (range, 50–74) for CTR patients and 63 months (range, 51–75) for UTR patients. A study flow chart and the patient demographic data are shown in Fig. 1 and Table 1, respectively.

**Table 1:** Patient demographics and outcomes

Variable	CTR^a^ (*n* = 132)	UTR^b^ (*n* = 136)	*p* value
Sex, M/F	68/64	65/71	0.542^c^
Age, years	67.43 ± 6.51	67.65 ± 6.13	0.351^d^
BMI, kg/m^2^	26.33 ± 5.14	26.84 ± 6.45	0.186^d^
BMD	− 3.64 ± 0.73	− 3.65 ± 0.29	0.125^d^
Side, left/right	74/58	70/66	0.451^c^
FNFs			0.486^e^
AO/OTA type 31B	93 (70.5)	101 (74.3)	
AO/OTA type 31C	39 (29.5)	35 (25.7)	
Comorbidities, *n* (%)			0.986^e^
Hypertension	35 (26.5)	31 (22.8)	
Diabetes mellitus	32 (24.2)	26 (19.1)	
Cerebrovascular accident	14 (10.6)	13 (9.6)	
Mechanism of injury, *n* (%)			0.330^e^
Traffic-related injury	32 (24.2)	35 (25.7)	
Injury by falling	73 (55.3)	82 (60.3)	
Tamp injury	27 (20.5)	19 (14.0)	
ASA index, *n* (%)			0.800^e^
I	36 (27.3)	35 (25.7)	
II	58 (43.9)	67 (49.3)	
III	38 (28.8)	34 (25.0)	
Preoperative HHS	58.37 ± 14.26	57.69 ± 16.31	0.182^d^
Follow-up period (months)	62.25 ± 12.13	63.43 ± 12.71	0.139^d^

*CTR* cemented total hip replacement, *UTR* uncemented total hip replacement, *HHS* Harris hip score, *ASA* American Society of Anesthesiologists, *BMI* body mass index, *BMD* bone mineral density, *FNFs* femoral neck fractures

^a^Exeter Universal stem combined with the All-poly cup (Stryker, Mahwah, NJ)

^b^Taperloc stem (Biomet, Warsaw, IN) combined with a ram-extruded bar stock polyethylene cup (GUR 415; Hoechst Celanese Corp, Houston, TX)

^c^Analysed using the chi-square test

^d^Analysed using an independent samples *T* test

^e^Analysed using the Mann–Whitney test

### Primary outcome

Table 2 lists the HHSs after treatment. From the 37 months postoperatively to the last follow-up, all HHS differences between the group of patients with FNFs (AO/OTA type 31B/C) undergoing primary CTR versus the group receiving primary UTR were significant, and CTR conferred a significant advantage in the HHS versus UTR for these patients. At each follow-up before the 37 months postoperatively, there were no significant differences between the groups. Almost 88% of the patients had an acceptable HHS at the last follow-up.

**Table 2:** Long-term follow-up: functional outcomes

Month(s) postoperatively	CTR^a^ (*n* = 132)	UTR^b^ (*n* = 136)	*p* value
1	80.22 ± 7.16	79.74 ± 8.32	0.261
3	83.86 ± 8.24	84.23 ± 9.51	0.206
6	88.46 ± 10.57	87.74 ± 11.29	0.157
12	89.36 ± 10.09	89.51 ± 10.26	0.132
24	87.43 ± 10.56	86.31 ± 11.21	0.127
36	85.21 ± 9.33	84.27 ± 8.84	0.102
37	84.77 ± 15.27	82.43 ± 16.15	0.045*
48	81.47 ± 17.29	78.41 ± 18.39	0.031*
49	81.22 ± 18.36	78.34 ± 19.23	0.029*
51	80.53 ± 19.26	76.45 ± 18.36	0.025*
54	80.12 ± 17.64	76.16 ± 16.65	0.022*
60	79.45 ± 17.36	74.67 ± 15.49	0.014*
Final follow-up	79.39 ± 16.92	74.18 ± 17.55	0.011*

*CTR* cemented total hip replacement, *UTR* uncemented total hip replacement, *HHS* Harris hip score

^a^Exeter Universal stem combined with the All-poly cup (Stryker, Mahwah, NJ)

^b^Taperloc stem (Biomet, Warsaw, IN) combined with a ram-extruded bar stock polyethylene cup (GUR 415; Hoechst Celanese Corp, Houston, TX)

*Statistically significant values

### Secondary outcome

**Fig. 2:**
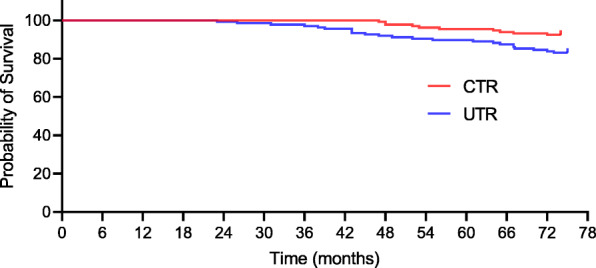
Kaplan–Meier survival curve for both groups with prosthesis revision for any reason as the endpoint

**Fig. 3:**
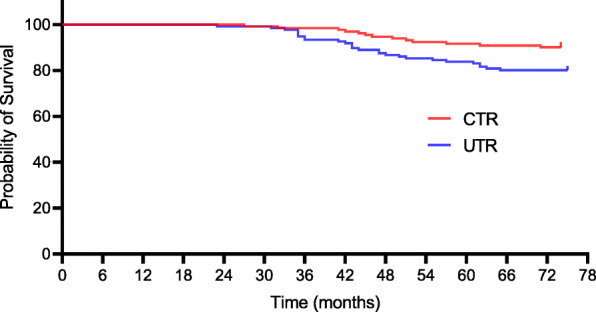
Kaplan–Meier survival curve for both groups with prosthesis loosening as endpoint

**Fig. 4:**
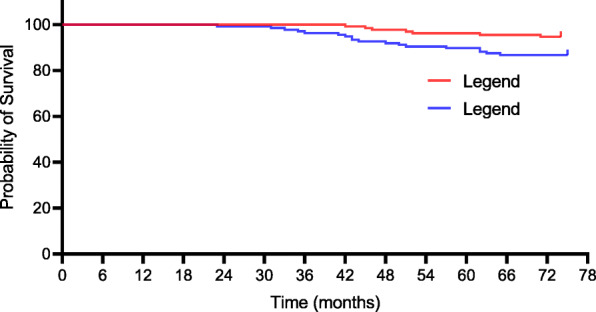
Kaplan–Meier survival curve for both groups with periprosthetic fracture as endpoint

At the final follow-up, seventy-two orthopaedic complications in 132 CTR-treated patients versus 111 orthopaedic complications in 136 UTR-treated patients were detected. Of the 72 complications observed in the CTR group, 10 (7.6%) required prosthesis revision, 13 (9.8%) were prosthesis loosening, and 7 (5.3%) were periprosthetic fractures. Of the 111 complications in the UTR group, 23 (16.9%) required prosthesis revision, 27 (19.9%) were prosthesis loosening, and 18 (13.2%) were periprosthetic fractures, as presented in Table 3. There was a noteworthy difference in terms of prosthesis revision, prosthesis loosening, and periprosthetic fracture at the last follow-up (7.6% [95% CI, 6.4–8.2] for CTR vs 16.9% [95% CI, 14.7–17.3] for UTR, *p* = 0.020; 9.8% [95% CI, 8.3–10.7] for CTR vs 19.9% [95% CI, 18.2–20.9] for UTR, *p* = 0.022; 5.3% [95% CI, 4.4–6.7] for CTR vs 13.2% [95% CI, 12.1–13.8] for UTR, *p* = 0.026, respectively). Kaplan–Meier survival curves for complications including prosthesis revision, loosening, and periprosthetic fracture were shown in Figs. 2, 3, and 4.

**Table 3:** Long-term follow-up: prosthesis-related complications

Variable, *n* (%)	CTR^a^ (*n* = 132)	UTR^b^ (*n* = 136)	*p* value
Prosthesis revision	10 (7.6)	23 (16.9)	0.020*^,c^
Prosthesis loosening	13 (9.8)	27 (19.9)	0.022*^,c^
Periprosthetic fracture	7 (5.3)	18 (13.2)	0.026*^,c^
Dislocation	6 (4.5)	9 (6.6)	0.461^c^
Femoral nerve palsy	3 (2.3)	4 (2.9)	0.732^c^
Insufferable hip pain	6 (4.5)	7 (5.1)	0.819^c^
Thrombotic events	2 (1.5)	0 (0.0)	0.242^c^
Heterotopic ossification	25 (18.9)	23 (16.9)	0.665^c^

*CTR* cemented total hip replacement, *UTR* uncemented total hip replacement, *HHS* Harris hip score

^a^Exeter Universal stem combined with the All-poly cup (Stryker, Mahwah, NJ)

^b^Taperloc stem (Biomet, Warsaw, IN) combined with a ram-extruded bar stock polyethylene cup (GUR 415; Hoechst Celanese Corp, Houston, TX)

^c^Analysed using the chi-square test

*Statistically significant values

## Discussion

This retrospective analysis characterized the long-term nationwide results of single brands of CTR or UTR in the Asian population and showed that CTR was superior to UTR in regard to the HHS and the orthopaedic complication rate. An important consideration when providing CTR or UTR is the potential for the high incidence of orthopaedic complications, given that CTRs may be associated with the highest number of orthopaedic complications in the context of FNFs (AO/OTA type 31B/C).

Our findings confirm and extend those of recent reports [6, 15, 22, 23]. Although the high HHS and the low incidence of orthopaedic complications tended to favour CTR, the HHSs did not differ significantly between the groups during the first 3 years of follow-up. The reasons for the discrepancy in clinical outcomes in patients with FNFs (AO/OTA type 31B/C) are unclear. Possible explanations include that the efficacy of CTR or UTR for improving clinical outcomes may stem from the dependence on time. When considering the clinical implications for CTR or UTR, it is important to comprehend the benefit-to-risk ratio for patients with FNFs (AO/OTA type 31B/C). When evaluating the impact of CTR on postoperative orthopaedic complications, we did not find an increased risk for other serious adverse results, irrespective of the presence of prosthesis revision or loosening. Published data [4, 19, 24] involving arthroplasties for FNFs have demonstrated that CTR can provide immediate strong interlocking between the implant and bone tissue and allows patients to carry out early weight-bearing activities. Nevertheless, these studies also point out that CTR is associated with a risk of cement-related adverse events (i.e. cardiopulmonary complications and bone necrosis attributable to heat during the polymerization of cement). The cement–bone interface can provide vulnerable conditions of infection [18]. This phenomenon requires further attention, as the performance of arthroplasty has been identified as a potential risk factor for adverse events [2]. Prosthesis revision is recognized as a catastrophic event and has been the focus of previous studies related to CTR or UTR [2, 4]. Our analysis showed that at the 3-year follow-up, neither group demonstrated evidence of prosthesis loosening or prosthesis revision, and there was no substantial distinction in the incidence of orthopaedic complications between the groups. However, it will be of utmost importance to explore whether cement resistance to bone microstructure exists for CTR after more than 3 years of follow-up and, if so, by what mechanism. Efforts to prevent prosthesis revision and the appropriate management of FNFs are important for improving clinical outcomes. Nevertheless, there remains no universally accepted standard for preventing prosthesis revision following CTR or UTR [11, 25].

The evidence in the literature regarding the preferred prosthesis that should be implemented for the primary treatment of FNFs (AO/OTA type 31B/C) is unclear [26, 27]. Furthermore, few studies have provided reproducible guidelines for avoiding mechanical complications [8]. A growing but still very limited body of literature has indicated that UTR is superior to CTR regarding functional outcomes with 1-3 years of follow-up [11, 12]. Nevertheless, in accordance with several multicentre, randomized reports [28, 29], we failed to detect between-group discernible differences in the HHS after 3 years of follow-up. This lack of difference could be due to the relatively short follow-up times. To our knowledge, there has always been controversy regarding functional outcomes [1, 2, 11], and the process of actually resolving these differences has been confounded by the differences in the number of subjects and the different follow-up times.

The SS of the proximal femur has been deemed to be inversely proportional to the diameter of the proximal femoral stem, and the occurrence of SS is universally recognized in UTR, although the long-term clinical importance of SS in UTR is unclear [30, 31]. The stiffness of press-fit prostheses with large diameters produces more SS and aggravates the resorption of the proximal femur around the prosthesis [30]. Hence, the high incidence of prosthesis revision that has been described in UTR is not unexpected and tends to be associated with differences in prosthesis materials [30, 32–34]. Studies such as these have resulted in controversy as to whether SS is merely a sign of bone resorption in this setting [30]. Experimental evidence indicates that SS can influence the microcirculation of bone tissue and that the magnitude of SS is clearly associated with different bone responses [33, 34]. Unfortunately, no observational trials have established causation between SS and prosthesis revision [31]. Undeniably, the current database does not allow us to unravel the potentially adverse events of SS-induced bone resorption from the potential advantages of UTR.

This study should be interpreted in light of several important limitations. First, one limitation of our study was the uncontrolled, retrospective study design, with certain questions inherent to the methodology. Confounders could have reduced the power to draw reliable conclusions, but well-matched cohorts permitted us to draw conclusions irrespective of the baseline characteristics. Second, simply excluding cases that did not have complete baseline data would have led to the introduction of substantial selection bias into the statistical analysis. Furthermore, the selection of surgical programmes among the patients was not randomized. Additionally, residual bias from confounding factors seemed to be inevitable due to certain unobtainable data.

## Conclusions

The results reported in our study support a growing body of evidence that CTR provides better functional outcomes and a lower incidence of orthopaedic complications than UTR in Asian patients with FNFs (AO/OTA type 31B/C). These findings may be conducive to mitigating ongoing discussions about the implementation of decision-making for surgery in these patients. Despite unavoidable limitations in this current study, our findings appear to be consistent with those of previous meta-analyses. Further follow-up may be necessary to verify whether our findings apply over the long term.

## Data Availability

The datasets used and/or analysed during the current study are available from the corresponding author upon reasonable request.

## Abbreviations

FNFs: Femoral neck fractures
CTR: Cemented total hip replacement
UTR: Uncemented total hip replacement
CT: Computer tomography
BMD: Bone mineral density
ISS: Injury severity score
COPD: Chronic obstructive pulmonary disease
BMI: Body mass index
ASA: American Society of Anesthesiologists
HHS: Harris hip score
CI: Confidence interval
SD: Standard deviation
IQR: Interquartile range
